# Presence of Dengue Virus NS1 antigen and IgM and IgG antibodies in asymptomatic individuals in Ecuadorian Amazonia - a descriptive repeated cross-sectional community-based survey

**DOI:** 10.1016/j.nmni.2026.101728

**Published:** 2026-02-19

**Authors:** Jacob van der Ende, M. Vanessa Davila, Josefina Coloma, Henk Schallig, Thomas Hanscheid, Martin P. Grobusch

**Affiliations:** a Hospital San Miguel, Fundación Quina Care Ecuador, Puerto El Carmen de Putumayo, Ecuador; b Center for Tropical Medicine and Travel Medicine, Department of Infectious Diseases, Division of Internal Medicine, Amsterdam University Medical Center, Location University of Amsterdam, Amsterdam, the Netherlands; c Instituto de Microbiología, Colegio de Ciencias Biológicas y Ambientales, Universidad San Francisco de Quito, Quito, Ecuador; d Division of Infectious Diseases and Vaccinology, School of Public Health, University of California, Berkeley, USA; e Laboratory for Experimental Parasitology, Department of Medical Microbiology and Infection Prevention, Amsterdam University Medical Centre, Amsterdam, the Netherlands; f Amsterdam Institute for Global Health and Development, Amsterdam, the Netherlands; g Amsterdam Institute for Immunology and Infectious Diseases, Amsterdam, the Netherlands; h Universidade de Lisboa, Faculdade de Medicina, Avenida Prof. Egas Moniz, 1649-028, Lisboa, Portugal; i Institute of Tropical Medicine and German Centre for Infection Research (DZIF), University of Tübingen, Tübingen, Germany; j Centre de Recherches Médicales en Lambaréné (CERMEL), Lambaréné, Gabon; k Masanga Medical Research Unit (MMRU), Masanga, Sierra Leone; l Institute of Infectious Diseases and Molecular Medicine (IDM), University of Cape Town, Cape Town, South Africa

**Keywords:** Dengue virus (DENV), NS1 antigen, Dengue IgM, Dengue IgG, Ecuadorian Amazonia, Malaria elimination

## Abstract

**Background:**

As malaria approaches elimination in Ecuadorian Amazonia, dengue virus (DENV) has emerged as the dominant vector-borne disease. A recent study documented circulation of DENV in the Putumayo northern border region, but could not determine whether transmission extended beyond road-connected areas into remote riverine communities. We conducted a community-based assessment to quantify dengue virus transmission across an urban–riverine gradient.

**Methods:**

This repeated cross-sectional community-based survey screened in total 293 asymptomatic individuals in four communities with, in total, an estimated 4.240 inhabitants along the Putumayo River during the 2023 rainy season. Participants were tested using an NS1/IgM/IgG combination rapid diagnostic test. Acute or recent infection was defined as NS1 and/or IgM positivity; past exposure as IgG-only positivity.

**Results:**

Overall, 8.5% (25/293) of asymptomatic participants had acute or recent DENV infection, and 28.7% (84/293) showed any serological evidence of exposure. The road-connected hub Puerto El Carmen had the highest total prevalence (50.9%), predominantly representing past infection (IgG-only: 42.5%). In contrast, remote riverine communities showed lower overall exposure but a higher proportion of acute/recent infections among positives—reaching 60% in Puerto Rodríguez—suggesting recent viral introduction into immunologically naïve populations.

**Conclusions:**

Dengue virus transmission in the Putumayo Amazonia region extends beyond hospital-detected cases into river-access-only communities. The inverted ratio of acute-to-past infection between urban and riverine settlements suggests source-sink dynamics with ongoing viral movement along fluvial networks. As malaria elimination progresses, integrated arboviral surveillance incorporating community-based RDT screening and field laboratories is essential to detect emerging viral transmission invisible to facility-based systems.

## Highlights

• In a serial cross-sectional community survey, 293 dengue-asymptomatic individuals in four communities along the Putumayo River were screened.
• Dengue transmission in the Putumayo region of Ecuadorian Amazonia extends beyond hospital-detected cases into communities only accessible by boat.
• The inverted ratio of acute to past infection between urban and riverine settlements suggests viral exportation along fluvial networks.
• Arboviral surveillance incorporating community-based RDT screening is essential to detect transmission invisible to hospital-based systems.

## 1 Introduction

The epidemiological landscape of vector-borne diseases in the Amazon basin is shifting rapidly. In 2024, the Region of the Americas reported almost 13 million dengue cases, the highest annual total on record [1]. In the Brazilian Amazon alone, DENV transmission expanded markedly between 2001 and 2021, with persistent high-risk clusters and multi-serotype circulation in riverine municipalities [2]. Over the same period, malaria declined, and border-focused research has begun to explore how malaria-oriented surveillance systems must adapt to emerging arboviral threats [3]. Ecuador and neighbouring countries combine ecological suitability, widespread *Aedes aegypti/albopictus* vector presence, and a high burden of arboviral disease, including yellow fever, Oropouche, Zika, chikungunya and especially dengue virus [[4], [5], [6], [7], [8], [9]]. Dengue predominates, accounting for approximately 91% of arboviral disease notifications over three decades, with all four serotypes present and DENV-1 and DENV-2 being most-common [8,10]. A recent population analysis of hospital deaths across 24 provinces of Ecuador between 2015 and 2023 counted 125 deaths due to dengue and 5 deaths due to malaria, with the highest number of deaths in Amazonia [11].

However, DENV transmission in Amazonian riverine systems is currently still under-appreciated as the difficulty to reach populations at risk in these difficult to reach remote regions has apparently not yet been recognized as a major case management and control challenge [12]. It includes a large ‘silent’ fraction that escapes routine surveillance: a systematic review showed that asymptomatic or mildly symptomatic infections frequently represent more than half of all dengue cases [13], and modelling indicates these infections drive much of onward transmission. Even clinically apparent disease is substantially under-captured: Brazilian evaluations document marked under-reporting of notified and hospitalised dengue [14]. In this same area, hospital data confirming co-circulation of all four DENV serotypes [10], suggesting a large reservoir of unrecognised infections in riverine communities, where asymptomatic cases, access barriers, and delayed referral create largely invisible outbreaks and could seed larger epidemics in urban centers.

Conventional diagnostics such as PCR and ELISA require laboratory infrastructure and cold-chain transport and are poorly suited to boat-access-only settlements. In contrast, commercial NS1 and NS1/IgM/IgG rapid diagnostic tests deliver results within minutes at the point of care, and are feasible for use in peripheral settings with limited equipment and training [15,16]. Integrated into surveillance or community surveys, these RDTs offer a pragmatic means of detecting probable dengue and beginning to quantify this hidden burden.

In Amazonia, rivers function as the principal transport corridors for both humans and vectors. Studies from Peruvian Amazonia demonstrate that *Aedes aegypti* is widely established along fluvial networks, transported by river boats, and human movement linking small riverine settlements with larger market towns, thus creating dense inter-community mobility structures that facilitate DENV spread [3,[17], [18], [19]]. Within this regional context, the Putumayo Amazon basin is a strategically important setting in which to characterise DENV transmission in a post-malaria, arbovirus-dominated environment. The Putumayo canton in the northeastern Ecuadorian Amazon reflects this pattern: historically a malaria transmission zone, it is now in a pre-elimination phase [11] with very low numbers of individuals with often asymptomatic parasitaemia [20]. Ecuador now reports circulation of all four dengue serotypes and dengue constitutes the majority of arboviral notifications [8,21]. Recent co-circulation of DENV-1 to DENV-4 among symptomatic patients at the regional reference hospital in Puerto El Carmen confirms the Amazon basin as an arboviral hotspot [10]. However, hospital-based surveillance captures only those able to reach clinical care and provides no information on infection or immunity within asymptomatic riverine populations. We therefore set out to estimate the extent of active or recent transmission across this urban–riverine gradient, to inform prioritisation for surveillance strengthening or targeted dengue vaccination in remote communities [[1], [2], [3],^8^,^10^,^20^].

## 2 Methods

### 2.1 Study design, setting, and participants

**Fig. 1:**
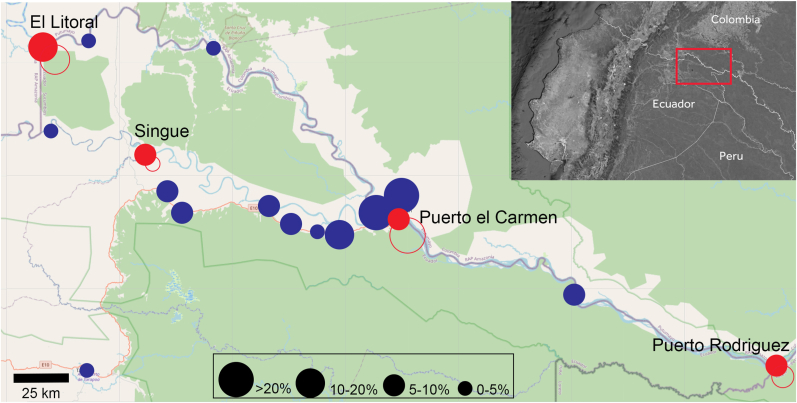
Study communities and acute and previous infections along the Putumayo River and San Miguel River, Ecuador. Map of the Putumayo canton in north-eastern Ecuador showing the four sampled communities (Puerto el Carmen, Singue, El Litoral, and the Puerto Rodríguez cluster) along the Putumayo River and San Miguel River. Red circle size indicates the percentage of asymptomatic participants sampled at each site (0-5%; 5-10%; 10-20%; >20%). Red closed circles indicate acute infection, red open circles indicate previous infection. Blue circle size indicates the number of symptomatic participants included in the previous hospital based DENV study. The inset shows the study area within Ecuador and adjacent border regions (Colombia/Peru).

We conducted a descriptive, repeated cross-sectional community survey in four communities in Putumayo canton, a remote Amazonian border region in northeast Ecuador, with data collection at three different timeframes in the same area. This work was carried out concomitantly with a malaria epidemiological investigation, with field procedures described elsewhere [20]. To capture seasonal variability, sampling was performed at three points during the 2023 rainy season—at the beginning (May), midpoint (August), and end (November–December), respectively. Four communities were purposively selected within the Putumayo canton to represent geographic variation and differences in accessibility along the Putumayo River corridor. El Litoral and the Puerto Rodríguez cluster were selected as border communities located at the western and eastern extremes of the canton, respectively. Puerto El Carmen, situated centrally within the canton, was included as the main urban settlement and transportation hub. Additionally, the community of Singue, located at the southwestern extreme of the canton, was included to extend spatial coverage beyond the primary river corridor (Fig. 1).

The study area shows marked contrasts in connectivity. Puerto El Carmen is the only sampled community with road access, forming the terminus of a paved regional route linking westward to Lago Agrio (Nueva Loja) the largest commercial center and onward to Colombia, and east–southeast toward La Joya de los Sachas and Coca. In contrast, El Litoral, Singue, and the Puerto Rodríguez cluster depend solely on the Putumayo River for transport, reflecting the characteristic isolation of Amazonian riverine communities and the epidemiological implications this entails for access to care, population mobility, and local transmission dynamics.

Following announcements by community leaders, participants were recruited through convenience sampling. Individuals aged three years or older who were asymptomatic at the time of sampling were eligible for inclusion. Participants were considered asymptomatic if they reported no fever (or history of fever within the preceding 48 h) and had no acute symptoms compatible with dengue infection (fever, rash, headache, malaise). Written informed consent was obtained from all adults, with parental or guardian consent provided for minors. Each sampling round was treated as an independent cross-section; participants presenting again within another timeframe were anew eligible for inclusion.

### 2.2 Sample collection and laboratory procedures

From each participant, a 3 mL EDTA blood sample was collected. Part of the sample was used for malaria diagnostics as described elsewhere [20]. On site, an INNOVITA NS1/IgM/IgG combo rapid dengue diagnostic test (INNOVITA (Tangshan) Biological Technology Co. Ltd., Hebei, China; Lot no. 20210701) was performed according to the manufacturer's instructions. The NS1/IgM/IgG combination format was selected to enable differentiation of the infection stage at point-of-care: NS1 antigen indicates acute infection with viraemia (typically detectable on days 1–7 of disease); IgM suggests recent infection (typically days 4–10 onwards); and IgG-only positivity indicates past exposure to flavivirus. This approach was chosen because standard IgG serology alone cannot detect acute asymptomatic dengue infections, yet quantifying this hidden viraemic fraction was a primary objective given its potential role in sustaining transmission cycles [15,16]. The manufacturer's package insert reports sensitivity of 95.6% and a specificity of 98.9%. Results were read independently by two trained field staff, with discrepancies resolved by a third reader. Invalid test results (absence of control line) were repeated immediately using the same sample.

Participants who tested positive for DENV by RDT were offered free medical advice and symptomatic treatment, including paracetamol, in accordance with local clinical practice.

### 2.3 Data analysis

Because of the low number of positive cases, no formal statistical testing was undertaken. Data were summarized descriptively.

### 2.4 Ethical clearance

Ethical approval was obtained from the Comité Ética de Seres Humanos of the Universidad Central, Quito (No. 414-CEISH-UCE-2023).

## 3 Results

### 3.1 Extent of active or recent dengue infections

A total of 293 asymptomatic participants were included and tested with the NS1/IgM/IgG combination RDT (Table 1). No exclusions were made based on the presence of symptoms. Among the 293 participants included, the mean age was 32 years; 33% were younger than 18 years, and 64% were male with similar demographics across all four sites. Overall, 25 participants (8.5%) had evidence of acute or recent DENV infection, defined as positivity for NS1 antigen, IgM antibodies, or both. Of these, 17 (68.0%) were IgM-positive in the absence of detectable NS1 antigen, consistent with recent rather than very early infection. Of these 25 participants with an acute infection, 3 (12%) participants were at the age of 5 years or younger, 3 (12%) were between six years and 18 years of age, 13 (52%) were between 18 years and 50 years of age, and 6 (24%) participants were older than 50 years of age. In addition, 59 participants (20.1%) were IgG-positive without detectable NS1 or IgM, indicating past DENV infection. Two participants were included at two time points. One of them was negative on both occasions, the other study case was IgG positive on both occasions. One participant was included on all three time points and tested negative on all three occasions.

**Table 1:** Dengue NS1/IgM/IgG rapid test profiles by community, Putumayo canton, Ecuador.

Location	Puerto el Carmen	El Litoral	Puerto Rodríguez	Singue	N_total_ of tests
Estimated population (n)	3100	30	1050	60	293
NS1- IgM- IgG-	52	25	101	31	209

NS1+ IgM- IgG-	1	2	1	1	5
NS1+ IgM + IgG-	0	0	0	0	0
NS1- IgM + IgG-	3	3	4	1	11
NS1+ IgM + IgG+	1	0	1	0	2
NS1+ IgM- IgG+	0	0	1	0	1
NS1- IgM + IgG+	4	0	2	0	6

NS1- IgM- IgG+	45	7	6	1	59

N_total_	106	37	116	34	293
% of total population	3.4	100*	11.0	56.7	6.9

Values are numbers of all asymptomatic participants (n = 293) tested with an INNOVITA NS1/IgM/IgG combination rapid diagnostic test NS1 = dengue virus non-structural protein 1 antigen; IgM = dengue virus–specific immunoglobulin M; IgG = dengue virus–specific immunoglobulin G; RDT = rapid diagnostic test; DENV = dengue virus. “Acute/recent infection” in the text refers to any profile with NS1 and/or IgM positivity (rows 2–7), whereas “past infection only” refers to IgG positivity in the absence of NS1 and IgM (NS1–/IgM–/IgG+). Puerto El Carmen is the only road-connected site; El Litoral, Puerto Rodríguez and Singue are riverine communities accessible only by boat. *Due to multiple sampling (N = 37) the number exceeds the number of the total population (123%). Numbers per round are in few cases duplicate, in one case triplicate.

Taken together, 84 of 293 participants (28.7%) showed serological evidence of past or current DENV exposure. NS1 antigen was detected in eight participants (2.7%), and 19 (6.5%) had IgM antibodies, either alone or in combination with NS1 and/or IgG.

### 3.2 Local dengue infection and immunity patterns

Evidence of past or recent infection varied markedly across the four study communities (Table 1). In Puerto El Carmen, the most urban riverside town and only road-connected site, nine of 106 participants (8.5%) had acute or recent DENV infection and 45 (42.5%) were IgG-positive without detectable NS1 or IgM, indicating past exposure. Overall, more than half of the participants in Puerto El Carmen (54/106, 50.9%) had serological evidence of past or current DENV infection, with the majority (83.3%) representing past rather than acute or recent infection.

Among the three road-inaccessible riverine settlements, overall exposure was lower but the composition differed. In El Litoral, 5/37 participants (13.5%) had acute or recent infection and 7/37 (18.9%) had IgG-only positivity (12/37; 32.4% with any DENV infection sero-marker). In Puerto Rodríguez, located near the junction of three countries (Ecuador, Colombia and Peru) along the river, 9/116 participants (7.8%) had acute or recent infection but only 6/116 (5.2%) were IgG-only positive (15/116; 12.9% with any marker). Notably, 60% (9/15) of DENV-positive individuals in this community had acute or recent rather than past infection, although this proportion is based on small absolute numbers and should be interpreted cautiously. In Singue, 2/34 participants (5.9%) had acute or recent infection and 1/34 (2.9%) had IgG-only positivity (3/34, 8.8% with any marker). The results corresponding to acute infections are summarized in Table 2.

**Table 2:** Details of communities visited regarding acute infections.

Community	Total inhabitants	N (%) of Community Sampled	N (%) of Acute Infections	N (%) of Previous Infections
**Puerto el Carmen**	3090	105 (3.4%)	9 (8.6%)	45 (42.9%)
**El Litoral**	30	37 (100.0%)	5 (14.5%)	7 (18.9%)
**Puerto Rodriguez**	1037	116 (11.2%)	9 (7.8%)	6 (5.2%)
**Singue**	58	35 (58%)	2 (5.7%)	1 (2.9%)

The community of Puerto Rodríguez comprises the combined localities of Tres Fronteras, Buen Samaritano, and Bajo Rodríguez.

These findings indicate a spatial gradient in overall DENV exposure, highest in the road-connected hub of Puerto El Carmen and lowest in the most isolated riverine communities. However, the ratio of acute or recent-to-past infection was inverted: Puerto El Carmen showed predominantly historical exposure, while the riverine communities, particularly Puerto Rodríguez, showed a higher proportion of recent infections relative to past exposure.

## 4 Discussion

The objective of this study was to estimate the extent of active or recent transmission across an urban–riverine gradient and implemented as an add-on to an existing community malaria surveillance platform. The study was not designed for longitudinal or temporal inference but to complement a hospital-based investigation that documented co-circulation of all four DENV serotypes around Putumayo during 2023–2024 [10]. In that study, clinical cases clustered almost exclusively along the national route EN 10, a paved interprovincial highway that ends in Puerto El Carmen and connects this small border town with Nueva Loja (Lago Agrio) and the wider Ecuadorian road network (Fig. 1). This road-associated pattern raised the question of whether transmission was largely confined to the EN 10 corridor or had also reached remote riverine communities accessible only by boat. The present findings suggest two contributing factors: dengue has reached the riverine settlements, but exposure remains substantially lower than in the road-connected hub (Fig. 1).

The spatial contrast is striking. In Puerto El Carmen, total serological exposure exceeded 50%, with most positives representing past infection (IgG-only), consistent with repeated transmission seasons in a well-connected urban node. The three riverine communities showed markedly lower overall exposure (9–32%) but a different composition: in Puerto Rodríguez, an important hub on a three-country point where many travellers stop over and do business, 60% of positives were acute or recent rather than past (Fig. 1). This inverted ratio suggests source–sink dynamics in which the road corridor serves as the transmission hub and riverine communities experience intermittent viral introductions rather than sustained endemic circulation [[17], [18], [19]]. Geographic remoteness appears to have provided partial, though incomplete, protection: transmission intensity in riverine settlements remains well below that observed in road-connected areas, yet the presence of acute markers confirms that isolation does not prevent spread once fluvial connectivity exists [22].

The detection of 8.5% acute or recent infection among asymptomatic individuals quantifies transmission invisible to hospital-based surveillance. More than half of DENV infections are clinically inapparent [13], and these silent infections drive onward transmission [23] because asymptomatic persons can still infect mosquitoes [24]. As data from Brazil suggest, routine surveillance may capture as little as 6% of confirmed cases [14], and in communities requiring hours of river travel to reach care, sensitivity is likely even lower. By contrast, sero-epidemiological studies from hyper-endemic Amazonian settings report much higher cumulative exposure: in Iquitos, Peru, neighbourhood-level dengue IgG seroprevalence commonly ranges between approximately 70% and 90% [25], and dengue IgG antibodies were detected in 41% of residents in riverine communities in Humaitá, Amazonas, Brazil [26], Against this background, the 20% IgG-only prevalence and 29% overall exposure observed in our riverine communities indicate that they remain relatively naïve, and therefore vulnerable to more severe outbreaks as multi-serotype circulation becomes established.

The predominance of IgM-only positivity (68% of acute/recent infections) over NS1 (2.7% overall) reflects survey timing rather than low transmission intensity. NS1 declines as antibodies rise, so cross-sectional screening of asymptomatic individuals preferentially captures later-phase infections where IgM persists but NS1 has been cleared [10]. The high proportion of asymptomatic infections may also reflect serotype-specific pathogenicity. In the companion hospital study, DENV-1 predominated among mild cases while DENV-2 was associated with more severe presentations [10]; if similar serotype distribution applied to our asymptomatic community sample - which we did not determine - this could partly explain the high proportion of subclinical infections observed.

The transition from malaria-dominated to dengue-dominated vector-borne disease burden observed in Putumayo mirrors a broader regional pattern [2,20]. As malaria elimination programmes succeed, arboviral diseases are filling the epidemiological space vacated by declining malaria transmission. Surveillance systems built around malaria, fever-triggered testing, microscopy-based diagnosis, treatment-focused algorithms, are poorly equipped to detect the often-subclinical transmission dynamics of arboviruses. Our data expose exactly this gap: DENV circulating largely undetected in communities that have exited the malaria surveillance system but have not yet entered any arboviral monitoring framework.

A key strength of this study is the community-based dengue data from riverine settlements in the Ecuadorian Putumayo canton, specifically designed to complement the earlier hospital-based investigation [10]. The clear road–versus–river gradient shown in Fig. 1, together with a standardised RDT protocol and double-reading, supports the internal consistency of our findings.

Limitations of this study include the use of a single commercial RDT without PCR or ELISA confirmation, recognizing that sensitivity is reduced in secondary infections and that it varies by serotype, as well as that cross-reactivity with other flaviviruses may inflate IgG estimates [15,16]. The INNOVITA NS1/IgM/IgG RDT used in this study carries CE marking and is manufactured under ISO 13485; however, no independent validation study of this specific product has been published. The NS1/IgM/IgG combination RDT format is endorsed by WHO interim guidance (2025) with pooled sensitivity and specificity of 91% and 96%, respectively [27]. Manufacturer-reported performance figures likely derive from symptomatic patients; sensitivity among asymptomatic individuals is expected to be lower, rendering our prevalence estimates conservative. In addition, a limitation of these RDTs is that they are calibrated to detect symptomatic dengue at relatively high viral loads; consequently, when used at a community level among individuals with mild or subclinical infections, they exhibit a high false-negative rate. Together with convenience sampling and modest sample sizes—particularly in Singue (n = 34) and El Litoral (n = 37)—that precluded stratified analyses and mean that proportional estimates such as '60% of positives in Puerto Rodríguez had acute/recent infection' (9/15) carry wide confidence intervals and should be interpreted cautiously. No systematic temperature measurement was performed, so asymptomatic status relied on self-report, participants were not tracked across rounds, preventing seroconversion assessment, and findings from four communities in one canton may not be generalizable.

From a public health perspective, DENV transmission in Putumayo has extended beyond the EN 10 road corridor into boat-access-only settlements with limited background immunity, populations vulnerable to future outbreaks as new serotypes circulate [8,21]. As single-dose dengue vaccines advance [28], heterogeneity between road-connected hubs and riverine communities highlights the need for local seroprevalence data and integrated surveillance strategies or emerging arboviruses reflecting the post-malaria epidemiological landscape.

These findings have direct implications for dengue control in the region. The marked heterogeneity between road-connected and riverine communities argues against uniform surveillance or vaccination strategies; instead, periodic community-based RDT screening at river transport nodes could detect transmission invisible to facility-based systems, while local seroprevalence data should guide prioritisation as new dengue vaccines become available.

## 5 Conclusions

This study demonstrates silent dengue circulation in a malaria pre-elimination setting in the Ecuadorian Amazon. Transmission has extended beyond the road-connected hub into riverine communities with low background immunity. As malaria approaches elimination, integrated arboviral surveillance—including RDT-based community surveys—is needed to detect transmission invisible to hospital-based systems.

## CRediT authorship contribution statement

**Jacob van der Ende:** Writing – review & editing, Writing – original draft, Methodology, Investigation, Formal analysis, Conceptualization. **M. Vanessa Davila:** Writing – review & editing, Investigation, Formal analysis. **Josefina Coloma:** Writing – review & editing, Writing – original draft. **Henk Schallig:** Writing – review & editing, Writing – original draft. **Thomas Hanscheid:** Writing – review & editing, Writing – original draft, Formal analysis. **Martin P. Grobusch:** Writing – review & editing, Writing – original draft, Methodology, Formal analysis, Conceptualization.

## Availability of data and materials

The datasets used and/or analysed during the current study are available from the corresponding author upon reasonable request.

## Funding

This study was funded in part by the Otto Kranendonk Foundation, Amsterdam, The Netherlands. In addition, it was supported by American Asian Centers for Arbovirus Research and Enhanced Surveillance (A2CARES) Grant 1U01AI151788 10.13039/100000002NIH/10.13039/100000060NIAID.

It was further supported by in-kind contributions from the Laboratory for Experimental Parasitology, Department of Medical Microbiology and Infection Prevention, and the Center for Tropical Medicine and Travel Medicine, both at Amsterdam University Medical Center, location 10.13039/501100003180Academic Medical Center at the 10.13039/501100001827University of Amsterdam, Amsterdam, The Netherlands.

## Declaration of competing interest

The authors declare that they have no known competing financial interests or personal relationships that could have appeared to influence the work reported in this paper.
